# Intraoperative and Postoperative Ultrasonographic Spinal Cord Evaluation of Cervical Double-Door Laminoplasty

**DOI:** 10.7759/cureus.61283

**Published:** 2024-05-29

**Authors:** Atsushi Matsuo, Kimiaki Sato, Kimiaki Yokosuka, Takuma Fudo, Koji Hiraoka

**Affiliations:** 1 Department of Orthopaedics, Kurume University, Kurume, JPN

**Keywords:** ossification of posterior longitudinal ligament, cervical spondylotic myelopathy, compression cervical myelopathy, double-door laminoplasty, percutaneous ultrasonography, ultrasonography

## Abstract

Background: Ultrasonography is a useful tool for the localization, morphology, and characterization of lesions and is increasingly being applied to spinal cord evaluation in cervical spine diseases. However, in conventional cervical laminoplasty, detailed evaluation is difficult because of ultrasound attenuation. Therefore, the purpose of this study was to perform a cervical laminoplasty using a modified surgical technique and evaluate the spinal cord.

Methods: The spinal cord was evaluated intraoperatively and one week postoperatively in 11 patients with cervical compressive myelopathy. Double-door laminoplasty was selected as the surgical method, and the shape and placement of the bone graft between the expanded laminas were devised to reduce ultrasonic attenuation, such that there was a large space in which the dura mater was visible.

Results: Intraoperative and postoperative spinal cord decompression, claudication, and pulsation were confirmed in all cases. A more precise diagnosis was possible using ultrasound attenuation using the grafted bone between the laminas as an indicator.

Conclusion: Intraoperative and postoperative spinal cord decompression status and wave patterns of modified cervical double-door laminoplasty can be evaluated using ultrasonography. Ultrasound-based evaluations of the spinal cord may provide new insights.

## Introduction

Intraoperative ultrasound evaluation was performed to localize spinal cord tumors and assess the decompression status of cervical compressive myelopathy [1-3]. Intraoperative dural pulsation, which can be confirmed using ultrasonography, is considered proof that the spinal cord is free within the subarachnoid space and is not under external compression [2,4]. Although magnetic resonance imaging (MRI) can depict spinal cord morphology and intramedullary conditions, dural pulsation is a finding that can only be captured using ultrasonography.

Previous studies have reported intraoperative observations of the spinal cord using ultrasonography and postoperative changes over time in cervical laminoplasty with suture anchor [5-7]. Similar to the Kirita-Miyazaki method [8,9], which is a type of laminoplasty, this method is advantageous for ultrasonography because there are no artificial bones, metallic materials, grafted bones, or other inclusions on the dorsal surface of the spinal cord, which do not cause attenuation of ultrasound waves.

At our hospital, double-door laminoplasty has been performed for compression cervical myelopathy since 1984, and the extracted spinous process is placed between the laminas [10]. In the present case, the shape and placement of the grafted bone were modified for the ultrasound diagnosis. Here, we present a surgical technique with this modification and report the results of intraoperative and postoperative ultrasonography. This study aimed to evaluate the feasibility of using ultrasound for spinal cord assessment in patients undergoing laminoplasty using a modified surgical technique.

## Materials and methods

The study was conducted at the Department of Orthopaedics, Kurume University, Kurume, Japan. The study was approved by the Ethics Committee of Kurume University (approval number: 22025). The inclusion criteria were patients diagnosed with cervical compressive myelopathy using examination, radiographic, computed tomography (CT), and MRI findings, who underwent laminoplasty surgery and had an ultrasound performed during surgery and one week postoperatively. The exclusion criteria were patients who did not have the diagnosis confirmed using radiography, CT, and MRI and patients who did not have an ultrasound performed during surgery or one week postoperatively. All the participants provided written informed consent to participate in this study.

This study involved 11 patients (eight males and three females) with cervical compressive myelopathy who underwent laminoplasty from April to June 2022, with a mean age of 74.2 (55-84) years. Eight patients had cervical spondylotic myelopathy, and three patients had ossification of the posterior longitudinal ligament. All patients had serious upper limb and/or lower limb disabilities that interfered with their daily lives.

Surgical techniques

**Figure 1:**
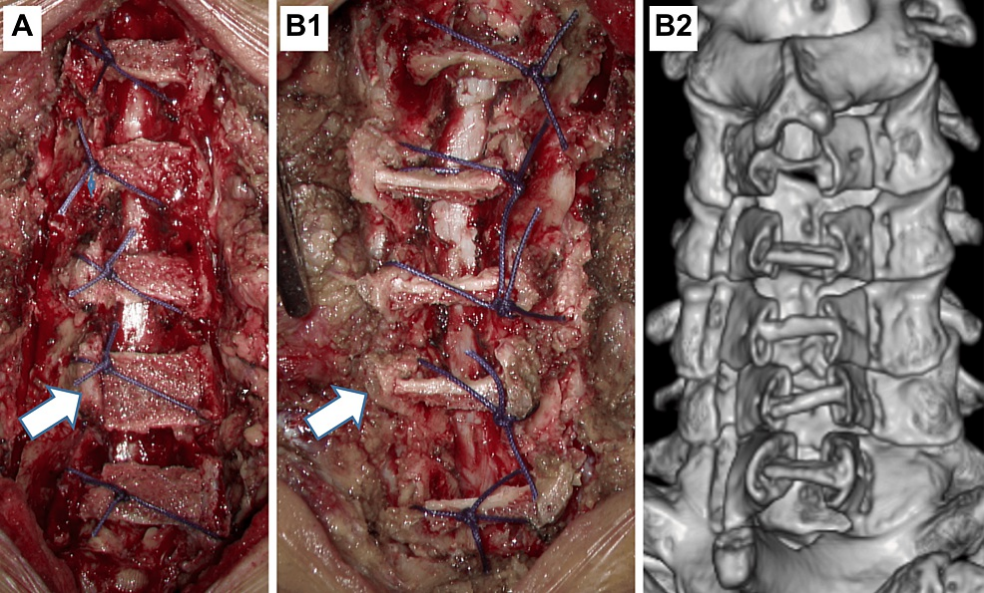
(A) Double-door laminoplasty with conventional surgical method. (B1) Double-door laminoplasty with modifications was performed. (B2) 3D-CT in the same case. The grafted bone was placed such that there was more space for visualization of the dura mater. 3D-CT, three-dimensional computed tomography

The patients underwent surgery under general anesthesia in the prone position, with the cervical spine in a forward bending position. All patients underwent laminoplasty at the C3-7 level. The autologous bone graft was placed perpendicular to the anterior forehead section to avoid reclosure of the expanded laminas and minimize ultrasound attenuation by the bone during postoperative percutaneous ultrasound examination (i.e., the graft was placed so that there was a large space between the graft and the bone to allow direct viewing of the dura mater) (Figure 1). After cleaning the wound, the subcutaneous tissue and skin were sutured, and the surgery was completed. All surgeries were performed by a single surgeon, and all steps were the same for patients with cervical myelopathy.

Ultrasound evaluations

Ultrasound examinations were performed intraoperatively and at one week postoperatively. All results were analyzed by two spine surgeons and no case was evaluated differently by the examiners. Intraoperative ultrasound examinations were performed using the immersion method (waterpath imaging technique) with the patient in the prone position. Two examinations were performed before and after the placement of the grafted bone between the expanded laminas. Percutaneous ultrasonography was performed in the sitting position one week after surgery. Intraoperative ultrasonography was performed using iViz Air (Fujifilm Holdings Corporation, Minato City, Tokyo, Japan) with a linear probe. Postoperative ultrasonography evaluation was performed using Xario 200 (Toshiba Corporation, Minato City, Tokyo, Japan) with a convex probe.

Spinal cord decompression status

The ventral subarachnoid space was evaluated intraoperatively and one week postoperatively, and spinal cord decompression status was classified into three grades according to Kowatari's classification [7], based on the level of contact. In Grade 1, or non-contact, the subarachnoid space remains retained ventral to the spinal cord within visual ultrasonographic range. In Grade 2, or non-contact and contact, the spinal cord was in contact with and separated from the posterior elements of the cervical spine. In Grade 3, or contact, the spinal cord was in continuous contact with the posterior elements of the cervical spine within the visible range.

Spinal cord pulse patterns

Spinal cord pulsation patterns were classified into six types using Nakaya's classification [11], ranging from no pulsation to a wave-like pulsation. In Pattern 0, there is no pulsation; in Pattern 1, there is a pulsating motion without displacement of the spinal cord; in Pattern 2, there is a pulsation as a sliding movement (forward and backward movement) of the entire spinal cord (cephalocaudal-caudal); in Pattern 3, there is a vertical movement (upward and downward movement) of the entire spinal cord (ventral-dorsal); in Pattern 4, there is a seesaw motion, different pulse cycles at the cephalic and caudal ends of the spinal cord; and in Pattern 5, there is a wave motion, a wave-like pulsation of the spinal cord.

The data were managed using Excel (Microsoft Corporation, Redmond, Washington, United States), but no statistical analyses were performed.

## Results

Intraoperative ultrasound evaluation clearly showed the subarachnoid space and spinal cord pulsation in all cases, both before and after placing the grafted bone between the laminas. The spinal cord decompression and pulsation patterns were identical in both cases. Furthermore, in all cases, the same intraoperative ultrasound findings were clearly observed at the one-week postoperative follow-up, despite the presence of postoperative asymptomatic epidural hematoma, swollen soft tissue, and subcutaneous and skin sutures.

There were no cases of worsening symptoms or intraoperative dural injuries that could affect the assessment of the spinal cord decompression status and pulse. Perioperative complications, such as intraoperative dural or neurovascular injuries, which could affect the assessment of spinal decompression status and pulse, did not occur. One case of surgical site infection (SSI) occurred, which was treated with the administration of antibiotics and surgical treatment.

Result 1. Decompression status

The number of patients according to decompression status over time is shown in Table 1. The number of grade 3 patients was zero, both intraoperatively and postoperatively. One week postoperatively, all patients decreased from grade 1 to grade 2.

**Table 1:** Change in the number of patients according to the decompression status over time

	Intraoperative (prone)	One week postoperative (sitting)
Grade1 (non-contact)	3	0
Grade 2 (non-contact and contact)	8	11
Grade 3 (contact)	0	0
Total	11	11

Result 2. Contact level between spinal cord and posterior vertebral elements

**Figure 2:**
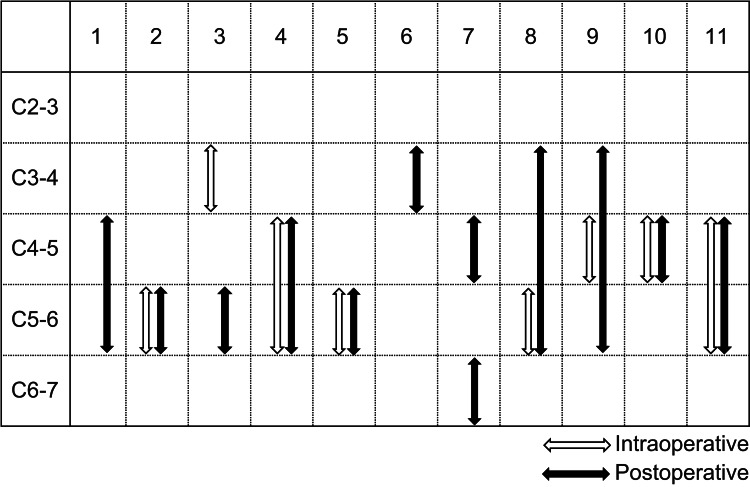
Contact heights between the spinal cord and posterior vertebral elements in each case (Cases 1–11)

Figure 2 shows the contact heights between the spinal cord and posterior elements of the vertebral bodies in each case. Eleven patients were observed and evaluated using ultrasound from C2-3 to C6-7. Intraoperatively, in the prone position, seven out of eight patients were in contact with the C4-6 level, and in the postoperative sitting position, 10 out of 11 patients were in contact with the C4-6 elevation. All seven cases with intraoperative contact at the C4-6 level remained in contact at the C4-6 level postoperatively. There were no cases of contact with the posterior elements of the vertebral body at the C2-3 level neither intraoperatively nor postoperatively.

Result 3. Pulse pattern

Table 2 shows the changes in the beating patterns over time. Spinal pulsation was observed in all patients intraoperatively and at the one-week postoperative follow-up. Pattern 1 was observed most frequently (five cases; 45.5%), both intraoperatively and one week postoperatively.

**Table 2:** Changes over time for each beat pattern

	Intraoperative (prone)	One week postoperative (sitting)
Pattern 0 (No pulsation)	0	0
Pattern 1 (Pulsating motion)	5	5
Pattern 2 (Forward and backward movement)	2	2
Pattern 3 (Upward and downward movement)	2	3
Pattern 4 (Seesaw motion)	0	0
Pattern 5 (Wave motion)	2	1
Total	11	11

**Figure 3:**
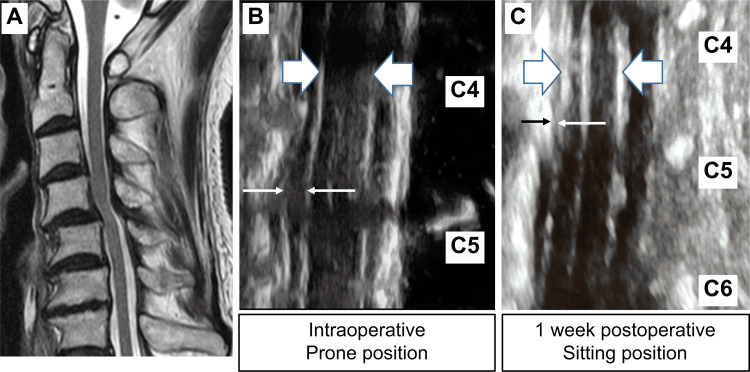
(A) Preoperative MRI, (B) intraoperative ultrasound findings, and (C) one-week postoperative ultrasonography findings of an 80-year-old male, diagnosed with cervical spondylotic myelopathy who underwent laminoplasty. The interior of ➡︎ (thick line) indicates the spinal cord, and the interior of → (thin line) indicates the ventral subarachnoid space. Intraoperatively, the ventral subarachnoid space was continuous, and decompression was grade 1. One week postoperatively, the spinal cord was in contact with the posterior elements of the vertebral body at the C4-5 level, and decompression status was Grade 2. The intraoperative pulsation pattern was pattern 5, “wave motion,”; however, postoperatively, a pattern 1 "pulsation" beat was observed.

Preoperative MRI, intraoperative ultrasound findings, and one-week postoperative ultrasonography findings of a randomly selected 80-year-old male participant diagnosed with cervical spondylotic myelopathy are shown in Figure 3.

## Discussion

In the conventional method of laminoplasty with a longitudinal split spinous process performed at our institution, it was found that the autogenous bone grafted between the expanded laminas must be of adequate length to increase the area of the spinal canal and placed parallel to the anterior foramina (i.e., the space between the graft and the grafted bone should be wide enough to allow a direct view of the dura mater). However, a certain amount of space is required to avoid interference with the mobility of the cervical spine. In addition, the contact area between the expanded laminas and grafted bone was widened from the viewpoint of bone fusion between the grafted bone and expanded laminas to prevent dislocation of the grafted bone. In addition, to prevent fracture of the grafted bone, appropriate thickness and width of the grafted bone were ensured to maintain sufficient mechanical strength. It is necessary to examine whether the new surgical method (with this modification) can achieve bone fusion in the same period as the conventional method because the contact area between the grafted bone and expanded laminas is small. It is also important, after accumulating more cases, to determine whether the expanded laminas will remain open because the grafted bone is fragile.

There is no consensus regarding the superiority of laminoplasty over laminectomy, although Henry et al. reported that adhesion to the dura mater and nerve root by the laminectomy membrane, which is present after laminectomy, can cause the recurrence of neurologic symptoms [12]. Some studies have suggested that laminectomy has a higher risk of kyphosis than laminoplasty [13,14]; however, others have reported no differences in postoperative outcomes between the two techniques [15]. Cervical double-door laminoplasty has been developing since the 1970s; although there are unresolved issues, it is widely used and there are many reports of its implementation [16-18]. However, laminectomy is more advantageous than conventional laminoplasty, in which an intervention exists on the dorsal surface of the dura mater to facilitate ultrasound examination. Few studies have investigated spinal decompression and pulsation using postoperative percutaneous ultrasonography [11,19]. The surgical technique presented here, in which the shape and placement of the grafted bone have been modified, allows postoperative spinal cord evaluation using ultrasound without major changes to the conventional technique, as well as higher-level diagnosis using the attenuation of the ultrasound by the grafted bone as an indicator.

In this study, intraoperative decompression status was the most favorable (Grade 1) in 3 cases (27.3%); nonetheless, 100% of cases were classified as Grade 2 at the one-week postoperative follow-up. Postoperative changes in decompression status are reportedly due to wound closure, muscle and soft tissue swelling, and asymptomatic epidural hematoma [11]. We believe that these local factors may be responsible for the early postoperative decompression changes observed as early as one week postoperatively in the current study. In addition, comparison and examination of ultrasonographic findings revealed that the spinal cord status changes depending on the position of the cervical spine [20]. Further, a report using ultrasonography showed that decompression status worsens when the patient is placed in a sitting position with the cervical spine in flexion and improves when the patient is placed in a supine position with the cervical spine in the mid-position [17].

Next, pattern 1, "pulsation," was the most frequently observed beating pattern in this study, both intraoperatively and at the one-week postoperative follow-up. Mihara et al. found that "sliding pulsation,” a phenomenon identical to pattern 2, "anterior-posterior pulsation,” was observed most frequently during cervical decompression surgery and also during the first postoperative week. These authors proposed that "sliding pulsation” of the spinal cord indicates proper decompression during cervical decompression surgery [3]. In this study, two patients (18.1%) had intraoperative "anterior-posterior pulsation" and Grade 2 intraoperative decompression, although it remains controversial whether an "anterior-posterior" pulsation pattern is considered adequate decompression even if Grade 1 decompression is not achieved. No study to date has examined the relationship between spinal cord pulsation patterns and cervical spine posture, which is a potential avenue for future research.

The advantage of performing postoperative percutaneous ultrasonographic spinal cord evaluation is that hematoma, soft tissue swelling, transplanted bone dislocation, and spinal cord pulsation can be easily assessed in the early postoperative period, and restenosis can be noted in the mid- to long-term. Although there were no cases of symptomatic hematoma requiring postoperative reoperation in this study, if the presence of a hematoma and the attenuation or disappearance of spinal cord pulsation can be evaluated at bedside, the time until hematoma removal can be shortened. In addition, with this technique, a more precise diagnosis can be made using the attenuation of ultrasound by the grafted bone as an indicator. The details of the "high position of the spinal cord in contact with the posterior wall of the vertebral body," which was demonstrated in this study, are still unknown, and it is hoped that the accumulation of future findings will lead to a better understanding of the pathophysiology of this condition.

Limitations

This study has several limitations. First, the number of cases was small and the observation period was short (one week postoperatively). In addition, ultrasonographic examinations were performed intraoperatively in the prone position and postoperatively in a seated position. The results might have been different if the same body position and cervical spine posture were used consistently. In addition, although all patients had compression myelopathy, the fact that cervical spondylotic myelopathy and ossification of posterior longitudinal ligament were included in the study, while not being the same disease, and that intraoperative and postoperative ultrasound examinations were not performed using the same model, may have influenced the results of the present study.

## Conclusions

Even with the longitudinally split spinous process of cervical vertebroplasty (a technique in which local bone is grafted between enlarged vertebral arches), it is possible to observe the spinal cord and surrounding tissues using ultrasonography by considering the shape and placement method of the grafted bone. Unlike MRI, this procedure can be performed at the bedside in real time and is considered a promising method for the postoperative evaluation of spinal disorders. The outcomes of the present study indicate that this is a powerful method for the postoperative evaluation of spinal disorders.
